# Relevance and application of sortase and sortase-dependent proteins in lactic acid bacteria

**DOI:** 10.3389/fmicb.2013.00073

**Published:** 2013-04-08

**Authors:** Emma K. Call, Todd R. Klaenhammer

**Affiliations:** Department of Food, Bioprocessing and Nutrition Sciences, North Carolina State UniversityRaleigh, NC, USA

**Keywords:** probiotic, sortase, sortase-dependent protein, LPXTG, lactic acid bacteria, *lactobacillus*, *lactococcus*

## Abstract

Lactic acid bacteria (LAB) are a diverse group of Gram-positive bacteria found in a vast array of environments including dairy products and the human gastrointestinal tract (GIT). In both niches, surface proteins play a crucial role in mediating interactions with the surrounding environment. The sortase enzyme is responsible for covalently coupling a subset of sortase-dependent proteins (SDPs) to the cell wall of Gram-positive organisms through recognition of a conserved C-terminal LPXTG motif. Genomic sequencing of LAB and annotation has allowed for the identification of sortase and SDPs. Historically, sortase and SDPs were predominately investigated for their role in mediating pathogenesis. Identification of these proteins in LAB has shed light on their important roles in mediating nutrient acquisition through proteinase P as well as positive probiotic attributes including adhesion, mucus barrier function, and immune signaling. Furthermore, sortase expression signals in LAB have been exploited as a means to develop oral vaccines targeted to the GIT. In this review, we examine the collection of studies which evaluate sortase and SDPs in select species of dairy-associated and health promoting LAB.

## INTRODUCTION

In Gram-positive bacteria, the cell wall is a crucial cellular component affecting a bacterium’s fitness and survival. The cell wall is responsible for maintaining structural stability, providing a barrier to osmotic pressures, and facilitating interactions with the surrounding environment. Cell walls of Gram-positive bacteria are decorated with a vast array of macromolecular structures which facilitate these interactions. These structures include teichoic acids, lipoteichoic acids, exopolysaccharides, Surface (S)-layer proteins, enzymes, and other cell surface proteins, such as adhesins and pili-like structures which are directly involved in host attachment (Marraffini et al., 2006; Weidenmaier and Peschel, 2008). The ecological niche of the microbes often dictates the mosaic-like surface display of macromolecules. The surface proteins of pathogenic microbes, such as internalin A in *Listeria monocytogenes *and protein A in *Staphylococcus aureus*, play a crucial role in establishing pathogenicity and infection (Mazmanian et al., 2000; Cabanes et al., 2002; Clancy et al., 2010). Alternately, in probiotic microbes, which confer health benefits upon the host, surface structures may play essential roles in eliciting these benefits.

Surface display is a twofold process composed of both protein targeting and protein attachment to the cell exterior. Protein targeting to the cell exterior is typically achieved through either the secretory (Sec) pathway or the twin-arginine translocation (TAT) pathway. The Sec pathway recognizes unfolded protein targets containing an N-terminal leader peptide, a hydrophobic core, and a C-terminal sequence that promotes binding of Sec machinery. Depending on the peptide sequence in the C-terminal region, the proteins are either exported out of the cell or N-terminally anchored in the membrane. Proteins that are N-terminally anchored in the membrane and processed by the Sec pathway represent a large proportion of membrane-anchored proteins in lactobacilli (Kleerebezem et al., 2010). Unlike the Sec pathway, the TAT pathway serves to transport folded protein to the cell’s exterior. This pathway appears to be much more uncommon in species of lactic acid bacteria (LAB). To date this pathway has only been identified in *Streptococcus thermophilus* and not in lactococci or lactobacilli (Hols et al., 2005). Further association of these proteins targeted to the membrane and cell exterior can either be achieved through covalent linkages or non-covalent interactions. The non-covalent interactions which may allow for protein association with the cell wall following export by Sec or TAT machinery have been reviewed (Schaffer and Messner, 2005; Kleerebezem et al., 2010).

**FIGURE 1:**
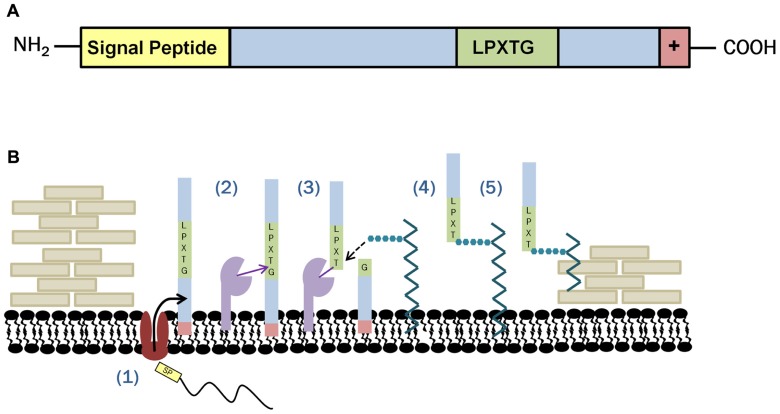
**Sortase anchoring in Gram-positive bacteria**. **(A)** Sortase substrates are recognizable due to the presence of an N-terminal signal peptide and a C-terminal LPXTG sorting signal followed by a series of hydrophobic and positively changed residues facilitating membrane anchoring. **(B)** Covalent linkage of sortase substrates to the cell wall is accomplished in a series of five steps: (1) Sec machinery recognizes the signal peptide on the sortase substrate and exports it to the cell’s exterior. The substrate remains embedded in the membrane due to the presence of a hydrophobic region terminated by a charged tail. (2) Once sortase and the sortase substrate are in proximity, sortase cleaves the target between the glycine and threonine residues via a transpeptidase reaction. (3) Nucleophilic attachment by lipid II disassociates the sortase/SDP complex and (4) forms a lipid II intermediate through interaction with the pentapeptide cross bridge. (5) In the final stages of sortase anchoring, the sortase substrate is incorporated into the cell wall as part of normal cell wall construction (adapted from Hendrickx et al., 2011).

One class of proteins covalently associated with the peptidoglycan of the cell wall after Sec targeting are the LPXTG-anchored proteins. These proteins contain a C-terminal cell wall sorting signal with the sequence of amino acids leucine (L), proline (P), X (representing any amino acid substitution), threonine (T), and glycine (G), and are linked to the cell wall by the housekeeping sortase, sortase A (SrtA). Successful linkage of sortase-dependent proteins (SDPs) to the cell wall is facilitated by the presence of not only the aforementioned LPXTG motif, but also a proceeding C-terminal hydrophobic region and a positively charged tail (**Figure 1A**). The N-terminal region of SDPs contains a signal peptide. This signal peptide enables secretion of the sortase substrate by the Sec pathway, while the C-terminal charged tail anchors the substrate once it reaches the cell membrane. Anchoring in the cell membrane by the C-terminal tail brings the SDP and the sortase enzyme, also embedded in the cell membrane, into proximity so that it may carry out the transpeptidation reaction required for cell wall anchoring. The first step in the transpeptidase reaction is the cleavage of the sortase substrate between the glycine and the threonine residue forming a sortase enzyme/SDP complex. The resulting thioester acyl bond between these two proteins is then subjected to nucleophilic attack and subsequent linkage to lipid II. Although lipid II is composed of both the peptidoglycan precursors, *N*-acetylglucosamine and *N*-acetylmuramic acid, as well as the pentapeptide peptidoglycan cross bridge, SDPs have been shown to link specifically to the pentapeptide (Maresso and Schneewind, 2008; Kleerebezem et al., 2010; Spirig et al., 2011). Once linked to the cross bridge, SDPs are incorporated into the cell wall with lipid II as it is translocated to the outer surface of the cell (**Figure 1B**).

Class A sortases which couple SDPs to the cell wall are the most well characterized; however, other classes of sortase enzymes have been identified. Class B sortases have been implicated in heme acquisition from the host, while class D, E, and F sortases have been identified and functionally analyzed to a lesser extent (Spirig et al., 2011). Class C sortases are better studied and play a critical role in pilus assembly. Class C sortases are transpeptidases, like class A sortases; however, they recognize a different sorting motif of (Isoleucine [I]/L)(P/Alanine [A]) XTG (Hendrickx et al., 2011). Functionally, class C sortases catalyze linkages between the Spa proteins to elongate the pilus shaft. Once elongation is complete, the pilus is anchored to the cell wall by either the class C sortase itself or by a class A sortase (Spirig et al., 2011). Similar to proteins coupled to the cell wall by class A sortases, the pili formed by class C sortases represent another mechanism of bacterial interaction with the environment. Pili are responsible for adherence to epithelial cells and extracellular matrix proteins, interaction with the host immune system, as well as biofilm formation (Danne and Dramsi, 2012).

Sortase proteins have been most extensively studied in the context of pathogens. SrtA was first identified in *S. aureus *where it is responsible for coupling between 18 and 22 substrates to the cell wall depending on the species (Marraffini et al., 2006). In *S. aureus*, as well as in *L. monocytogenes *and other Gram-positive pathogens, the deletion of the gene encoding the sortase enzyme showed attenuated virulence in animal models (Mazmanian et al., 2000; Bierne et al., 2002; Garandeau, 2002). This decrease in infective capability is attributed to the loss of adhesins, internalins, clumping factors, or host evasion molecules such as protein A from the cell surface of *S. aureus*. Not surprisingly, the product of sortase C action, the pilus, has also been implicated in pathogenicity through adhesion and host cell invasion in, but not limited to, *Corynebacterium diphtheriae*, *Streptococcus pneumoniae*, *Streptococcus pyogenes*, and *Actinomyces naeslundii* (Ellen et al., 1978; Ton-That and Schneewind, 2003; Mora et al., 2005; Barocchi et al., 2006; Gaspar and Ton-That, 2006; Telford et al., 2006; Mishra et al., 2007). Taken together, these findings were suggestive that sortase inhibition could function as an anti-infective therapy as thoroughly reviewed by Maresso and Schneewind (2008).

Sortase enzymes are found in all Gram-positive microbes, including food grade and health-relevant microbes of the LAB. Members of the LAB have a history of safe use and consumption of these microbes has been associated with health benefits, including competitive inhibition of pathogens, maintenance of epithelial barrier function, and a reduction in the symptoms of irritable bowel syndrome (Ventura et al., 2009). Nonetheless, in many cases the mechanisms associated with these effects are unknown. The sortase enzyme and SDPs in LAB are of interest in delineating the molecular mechanisms of host-bacterial interaction. While SrtA enzymes have been identified in a handful of LAB members, the sortase C enzyme has only been functionally characterized in *Lactobacillus rhamnosus *GG (Kankainen et al., 2009). Furthermore, the sortase cell wall anchoring machinery in LAB has been explored in the development of vaccines which could be administered orally as strains generally recognized as safe (GRAS).

## COMPARATIVE ANALYSIS OF SORTASE IN LAB

As previously stated, the sortase protein is ubiquitous among Gram-positive bacteria, and members of the LAB family are no exception. Sequencing of various LAB genomes has allowed for the identification of bacterial genes, including those that code for sortase. In 2002, a collaborative effort between the Department of Energy – Joint Genome Institute and the scientists of the Lactic Acid Bacteria Genome Consortium announced an elaborate sequencing project which aimed to make LAB genomes available to the public. Four years after the announcement of the initiative, 18 genomes of LAB were publically available (Makarova et al., 2006). Approximately a decade later, over 26 LAB genomes are available (Zhou et al., 2010). Encompassed in this collection of LAB are probiotic strains which include *Lactobacillus acidophilus*, *Lactobacillus gasseri*, and *Lactobacillus plantarum*. The genome assemblies have shed light on the presence of cell surface-associated structures which are suggested to modulate the microbe–host response. 

**FIGURE 2:**
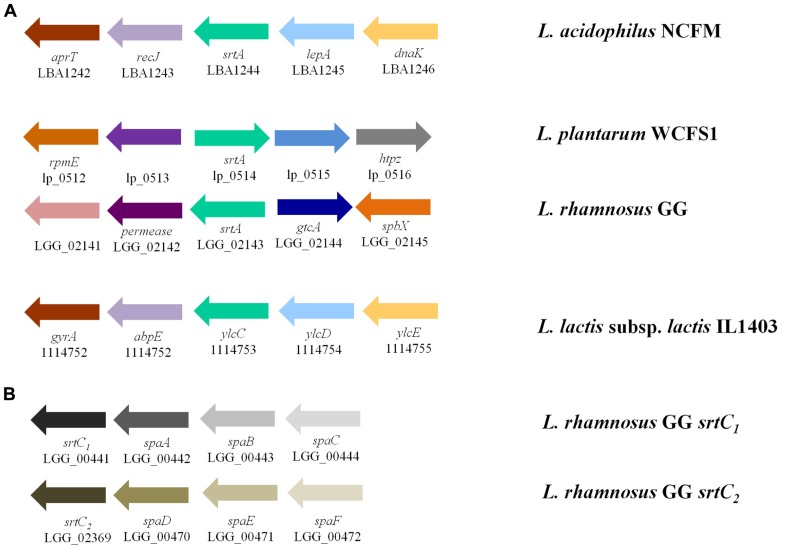
**Genomic context of genes encoding sortases in LAB**. **(A)** Genomic context of sortase A (green arrow) as found in *L. acidophilus *NCFM, *L. plantarum *WCFS1, *L. rhamnosus *GG, and *L. lactis *subsp*. lactis *IL1403 vary greatly. The sortase in *L. lactis *is not yet annotated as such; however, the gene annotated *ylcC *was identified to be the putative sortase enzyme by Dieye et al. (2010). These species were chosen to represent species from members of the acidophilus complex, other probiotic lactobacilli, and food-associated LAB. **(B) **Unlike sortase A whose targets are dispersed throughout the genome, sortase C enzymes cluster with their pili-subunit targets (adapted from Kankainen et al., 2009)

**FIGURE 3:**
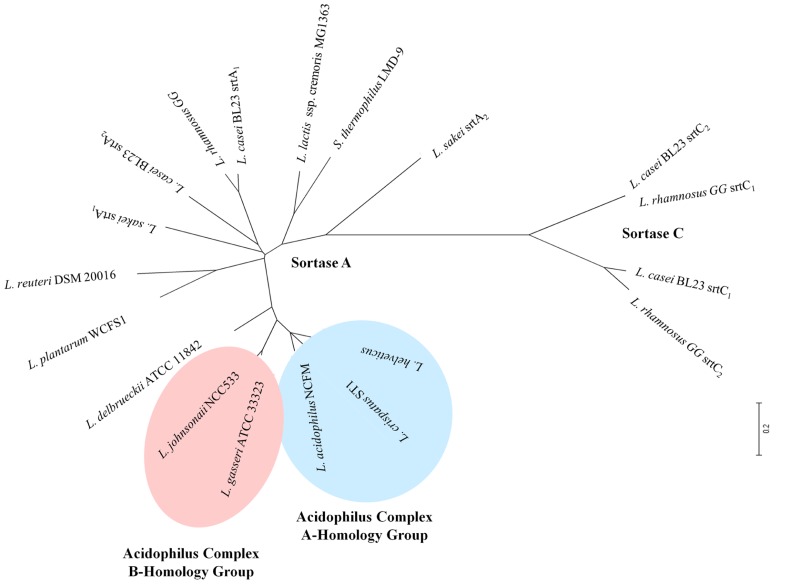
**Unrooted phylogenetic tree of sortase enzymes in different LAB**. Sortase enzymes were identified using available genome sequences in the NCBI database. Protein sequences were aligned in ClustalX v.2 and then imported into MEGA 4 for phylogenetic tree construction. Blue shading is used to highlight those LAB which are members of the acidophilus A-homology group, while light red shading is used to highlight those members of the B-homology group.

The role of sortase enzymes in the attachment of proteins to the cell wall makes it an attractive target for genome mining of structures involved in bacterial-host interactions. Genes encoding housekeeping sortase enzymes (*srtA*) have been identified in the genomes of LAB including *L. acidophilus *(Buck et al., 2005), *Lactobacillus salivarius *UCC118 (van Pijkeren et al., 2006), *Lactobacillus johnsonii *NCC533 (Denou et al., 2008), *L. rhamnosus *GG (Kankainen et al., 2009), *Lactococcus lactis *IL1403 (Dieye et al., 2010), *Lactobacillus casei *BL23 (Munoz-Provencio et al., 2012), *L. plantarum *(Remus et al., 2013), and *Lactobacillus crispatus *ST1 (Edelman et al., 2012). Additionally, genes encoding class C sortase proteins (*srtC*) have been identified in both *L. rhamnosus *GG (Kankainen et al., 2009) and* L. casei *BL23 (Munoz-Provencio et al., 2012). The genomic context of the *srtA *locus varies widely among species (**Figure 2A**); however, *srtC *genes have been found to cluster with their targets (*spa *genes) as was observed in both *L. rhamnosus *GG (**Figure 2B**) and *L. casei *BL23 (Munoz-Provencio et al., 2012). An unrooted phylogenetic tree shows the relationships between the sortase protein sequences identified in the aforementioned species of LAB as well as those present in other LAB species including *L. lactis *subsp. *cremoris *MG1463 and *S*. *thermophilus *LMD-9 (**Figure 3**). As expected based on differences in target proteins, distinct clusters formed indicating divergence in amino acid sequences of the housekeeping sortase, SrtA, and the pilin sortase, SrtC. Additionally, the SrtA proteins from group A members of the acidophilus complex cluster independently from those of the group B acidophilus complex, and those LAB (i.e.,* L. lactis *and *S. thermophilus*) whose 16s rRNA are divergent from those of lactobacilli also possess SrtA enzymes which cluster independently.

Further analysis of the genome content of these species allows identification of those substrates that sortase covalently links to the cell wall. SDPs can be identified based on the presence of a C-terminal LPXTG motif as well as an N-terminal signal peptide followed by a series of hydrophobic residues, as previously discussed. SDPs were first identified in sequenced genomes using from a hidden Markov model which predicted 732 sortase targets in 49 different prokaryotes (Boekhorst et al., 2005). Years later, those LPXTG containing proteins found in prokaryotic LAB were compiled and combined with other cell wall anchored and secreted structures to form the LAB secretome database^1^ (Zhou et al., 2010). This database currently contains 26 LAB and is publically available to aid in the identification of SDPs as well as other secreted structures. Although the software is capable of identifying SDPs, it is unable to distinguish between those SDPs which are pseudo or truncated genes, and those SDPs which are fully functional genes containing the three domains described above. The presence and of the signal peptide and other protein domains can be determined using InterProScan^2^, while the functionality and cleavage site within the signal peptide can be determined using SignalP 4.0 software^3^. A combination of these tools and a survey of the current literature was used to develop a table summarizing the prevalence of sortase enzymes, SDPs, and the functionality of SDPs in some common LAB (**Table 1**).

**Table 1:** Comparative genomic analysis of sortase-dependent proteins in select species of LAB.

Bacterial Species	Sortase protein	Presence (+) or absence (-)	Number of targets^a^	Predicted functional targets^b^	Reference(s)
*L. salivarius* UCC118	srtA srtC	+ -	10 0	4 0	van Pijkeren et al. (2006)
*L. casei* BL23	srtA	+ (2) + (2)	17 6	13 5	Munoz-Provencio et al. (2012)^d^
*L. plantarum *WCFS1	srtA srtC	+ -	32 0	27 0	Remus et al. (2013)
*L. lactis *IL1403	srtA srtC	+ +	11 3	9 Not expressed under normal conditions	Dieye et al. (2010) Oxaran et al. (2012)
*L. rhamnosus *GG**	srtA srtC	+ + (2)	Unknown^c^ 6	N/A 6	Kankainen et al. (2009) and von Ossowski et al. (2010)
*L. gasseri *ATCC 33323	srtA srtC	+ -	12 0	7 0	Kleerebezem et al. (2010)
*L. acidophilus* NCFM	srtA srtC	+ -	12 0	8 0	This review

a Targets containing an LPXTG motif were predicted using the hidden Markov model described by Boekhorst et al. and are now currently compiled in the LAB secretome database (Boekhorst et al., 2005; Zhou et al., 2010).

b Functional targets were determined based on the presence of a functional signal peptide (described in the literature or as determined by SignalP 4.0) and a LPXTG or LPXTG-like motif.

c *L. rhamnosus *GG is not a part of the LAB secretome database developed by Zhou et al., 2010.

d Twenty three total SDPs were identified and described by this group. In this review, predicted SDP’s were further clarified based on the targeting by SrtA or SrtC as well as their predicted functionality based on the parameters above.

## FUNCTIONAL ANALYSIS OF THE HOUSEKEEPING SORTASE AND SORTASE-DEPENDENT PROTEINS (SDPs) IN LAB

Prior to genome mining for SDPs, Roos and Jonsson (2002) first described the function role of a protein in the SDP family in *Lactobacillus reuteri *1063. The previously uncharacterized putative cell surface protein (NCBI reference sequence: AF120104) contained repeat regions homologous to mucus-binding (mub) domains and a C-terminal sortase recognition LPQTG motif. Furthermore, the protein contained an N-terminal secretion signal consistent with the conserved structure of functional sortase targets. The protein was called “Mub” and recombinant forms of the protein showed adherence to mucin derived from different animal species (Roos and Jonsson, 2002). Although the publication did not identify a sortase protein itself, the genome sequence of a different strain of *L. reuteri *(DSM 20016) with an available genome indicated the presence of a sortase enzyme (NCBI reference sequence: YP_001270843.1). This study asserted the first suggestion of an adhesive protein in a *Lactobacillus *species, and furthermore, the first suggestion that LPXTG-anchored motifs may play a role in mucus-binding in the gastrointestinal tract (GIT) of the host.

Similar adhesion factors were identified in *L. acidophilus *NCFM after its genome sequence became available in 2005 (Altermann et al., 2005). Three proteins, LBA1633, LBA1634, and LBA1392, were identified for their putative adhesive capacity based on their sequence homology with R28 protein from *S. pyogenes *(Buck et al., 2005). In *S. pyogenes, *R28 plays a role in binding to a cervical epithelial cell line (Stalhammar-Carlemalm et al., 1999). Additionally, the *L. acidophilus *protein, LBA1392, was found to have 25% protein homology to the Mub identified in *L. reuteri *(Buck et al., 2005). Interestingly, all three of these proteins contained a C-terminal LPXTG sorting signal. The presence of the LPXTG signal in these three putative Mubs as well as in nine other open reading frames in the *L. acidophilus *genome were suggestive of the presence of a sortase protein identified as LBA1244. Deletion and functional analysis of the sortase linked Mub, LBA1392, showed significant impairment in adherence to humans Caco-2 epithelial cells, but this observation was not reproducible for LBA1633 or LBA1634 (Buck et al., 2005). The nine other predicted sortase targets remained uninvestigated in this study.

The aforementioned studies in *L. reuteri *and *L. acidophilus *NCFM implicated surface proteins in specific adhesive roles of probiotic bacteria in the GIT and directed attention to the sortase protein and SDPs as an important mediator of this positive probiotic attribute. Further comparative analysis of cell surface structures associated with probiotic bacteria revealed that LPXTG-anchored targets could be found in common probiotic species including *L. plantarum *WCFS1, *L. johnsonii *NCC 533, *Lactobacillus sakei *23K, and *L. salivarius *UCC118 (**Table 1**). Furthermore, the number of targets present varied from species to species, with the highest number found in *L. plantarum *WCFS1 (Kleerebezem et al., 2010). While the role of sortase and SDPs in *L. plantarum *was evaluated at a later time (Remus et al., 2013), the initial focus was placed on determining the functional role of sortase and SDPs in *Lactobacillus salivarius *UCC118. Publication of its genome revealed the presence of ten SDPs consisting of four intact targets and six pseudogenes (Claesson et al., 2006; van Pijkeren et al., 2006).

With the genome sequence available, functional analysis of sortase and SDPs in *L. salivarius *UCC118 were greatly expedited. The sortase gene, identified as LSL_1606, was deleted while the four functional SDPs were insertionally inactivated (van Pijkeren et al., 2006). In two separate *in vitro *adhesion assays, one using Caco-2 and one using HT-29 adenocarcinoma epithelial cells, the sortase-deficient strain showed significant decreases in adhesion. One of the SDPs, named LspA (LSL_0311), was shown to contribute to this phenotype, while two others (LspB and LspD) did not appear to significantly reduce adhesion in these model systems. Not surprisingly, the SDP LspA contained a series of mucus-binding domain as were previously described as involved in adhesion in both *L. reuteri *and *L. acidophilus *NCFM (van Pijkeren et al., 2006). Current annotations of this protein describe it as Mbp2 since Lsp is used to denote lipoproteins structures unrelated to sortase.

A collection of SDPs from other probiotic microbes, including the mannose-specific adhesin (msa) in *L. plantarum *299v (Gross et al., 2008) and the *Lactobacillus *epithelial adhesin (LEA) isolated from *L. casei *BL23 (Edelman et al., 2012) have been shown to contribute to bacterial adhesion. In *L. casei *BL23, twenty three SDPs were predicted; however, none were specifically targeted for investigation. Alternately, each of the four sortases genes (two *srtA *and two *srtC*) were inactivated and the adhesion phenotype examined. In this study, inactivation of both SrtA enzymes was required to functionally impact binding to colonic epithelial cell lines, while inactivation of either of the class C sortases did not impact adhesion (Edelman et al., 2012). The latter finding was unexpected since in *L. rhamnosus *GG this gene is essential in constructing pili which directly contribute to epithelial cell adhesion (Kankainen et al., 2009). It is unclear which of the twenty three predicted SDPs plays a specific role in the adhesion phenotype as none share homology with Mubs found in *L. acidophilus *or *L. reuteri *(Edelman et al., 2012). However, one SDP (LCABL_23040) shares homology with a mucus-binding factor (MBF) in *L. rhamnosus *GG and may provide insight into SDPs mediating adhesion in *L. casei *BL23 (Munoz-Provencio et al., 2012).

Recent interest in sortase and SDPs has begun to focus on the role of these proteins in the immunomodulatory capacity of probiotic bacteria. These bacteria have the capacity to influence immune signaling of the colonic epithelium directly and through modulation of NF-κB signaling pathways. As an example, *L. rhamnosus *GG and *L. plantarum *BFE have been shown to enhance innate immune signals by increasing the expression of Toll-like receptors (TLRs) in HT-29 cells (Pinto et al., 2009). Additionally, antagonists of the potent pro-inflammatory transcriptional regulator, NF-κB, were up-regulated after one hour exposure of Caco-2 cells to *L. acidophilus* NCFM based on microarray analysis (O’Flaherty and Klaenhammer, 2012). This same technique was used to investigate transcriptional responses of Caco-2 cells exposed to both the sortase-deficient mutant of *L. salivarius *UCC118 and the wild type (O’Callaghan et al., 2012). Although the wild type strain showed down regulation of the NF-κB antagonist as well as induction of some innate immune regulators such as chemokines, the immune signaling pathways did not appear to be different after exposure to ∆*srtA L. salivarius *UCC118. However, epithelial mucin genes were significantly down-regulated after exposure to ∆*srtA L. salivarius *UCC118 (O’Callaghan et al., 2012). Mucin is important in the colonic epithelium for maintaining lubrication and barrier functionality of the GIT as well as preventing pathogen penetration into epithelial cells of the GIT (Shirazi et al., 2000; Moran et al., 2011). Down-regulation of the mucin genes after exposure to *L. salivarius *UCC118 lacking sortase and thus SDPs, implicate SDPs in stimulating mucin production to maintain barrier function as well as in adhesion, as previously described (van Pijkeren et al., 2006).

The role of sortase in non-pathogenic species has been focused, but not limited to, probiotic LAB. Initial studies using lactococci as a vaccine carrier suggested sortase machinery was functional due to the ability to display known sortase-anchored proteins from other species (e.g., M6 from *S. pyogenes*) on the surface of *L. lactis *ssp. *cremoris* (Norton et al., 1996; Piard et al., 1997). Additionally, amino acid sequence comparison of the *L. lactis *ssp. *cremoris *genes including the sex-factor aggregation gene (*cluA*) and proteinase P (PrtP) showed regions of homology to the LPXTG domains of cell wall-anchored proteins (Vos et al., 1989; Godon et al., 1995). This provided evidence for localization of PrtP at the cell wall and its display outside the cell envelope in a location important for nutrient acquisition in a dairy environment (Vos et al., 1989). Further investigation into sortase machinery in the industry-relevant strains has been accomplished in *L. lactis *ssp. *lactis *IL1403. This specific *L. lactis *has two sortase genes, *srtA *and *srtC*. The *srtC *gene is only expressed at low levels and does not appear to build functional pili under normal growth conditions (Oxaran et al., 2012). In contrast, SrtA couples at least five proteins to the cell wall of *L. lactis *ssp. *lactis *IL1403, some of which were shown to contain mucus-binding domains homologous to those present in some lactobacilli. This finding is suggestive of potential binding capacity to cellular components of the human GIT although this has not been demonstrated experimentally. In addition, PrtP is not found in this particular species although it can be found it *L. lactis *ssp. *cremoris *MG1363 (Dieye et al., 2010).

Variation in the sortase gene in species of *S. thermophilus *can also be observed. For example, the genome of *S. thermophilus *LMD-9 appears to encode an intact sortase, while the genomes of *S. thermophilus *CNRZ1066 and *S. thermophilus *LMG 13811 both harbor truncated enzymes (Goh et al., 2011). Furthermore, the latter two species do not contain genes encoding SDPs (Bolotin et al., 2004), while *S. thermophilus *LMD-9 potentially contains three SDPs, as predicted by the LAB secretome database (Zhou et al., 2010; Goh et al., 2011). In the case of *S. thermophilus *CNRZ1066 and *S. thermophilus *LMG 13811 this further substantiates the hypothesis of genomic decay during adaptation to milk and loss of gene features, including cell surface proteins, shown to contribute to virulence in related streptococcal pathogens (Bolotin et al., 2004).

This collection of studies represents the state of functional analysis of the housekeeping SrtA and its targets in probiotic species of lactobacilli as well as the limited investigation of this enzyme in food-associated *L. lactis *and *S. thermophilus*. While the studies published on SrtA in probiotic lactobacilli are not exhaustive, they suggest an important role for this enzyme and SDPs in adhesion to the intestinal mucosa of the host. Moreover, the functional role of sortase in adhesion is predicted to be mediated through its role in linking mucus-binding proteins or similar protein structures, such as the MBF found in *L. rhamnosus *GG (Munoz-Provencio et al., 2012), to the cell wall. Additionally, although not investigated in great detail at this point in time, the adhesive capacity of different probiotic strains to mucus and the GIT may function to allow interactions with the local immune system in the GIT. Beyond investigating gene expression of epithelial cell lines such as Caco-2 cells after probiotic exposure, it may be prudent to explore the responses of dendritic cells (DCs) to such treatment. DCs are resident immune cells in the GIT with the capacity to sample antigens and signal the immune system through cytokines. This approach to investigating immune stimulation by probiotic bacteria has been employed with regard to a sortase-deficient mutant of *L. plantarum *WCFS1, although significant changes in the amounts of anti-inflammatory IL-10 and pro-inflammatory IL-12p70 were not detectable when DCs were cultured with the individual strains (Remus et al., 2013). Studies such as these with other strains, both wild type and sortase-deficient, will help unravel the mechanisms behind probiotic functionality. Namely, as suggested by O’Callaghan et al. (2012) the combined adhesive capacity provided by some SDPs and the immune stimulation induced by probiotics, acting together, may condition the GIT for potential pathogen exposure. 

## THE PILI SORTASE: CHARACTERIZATION OF SrtC IN *L. rhamnosus* GG

Like the housekeeping sortase, originally discovered in the Gram-positive pathogen *S. aureus*, the sortase responsible for pili assembly, sortase C, was first described in the pathogen *C. diphtheriae *(Ton-That and Schneewind, 2003). It was shown to play a key role in assembling subunits, namely SpaA and SpaC, to form the pili found to protrude from the surface of *C. diphtheriae*. Pili are filamentous structures, approximately 1–2 μm in length, and usually numerous in their display on the bacterial surface. Since their discovery in *C. diphtheriae*, pili have been described in many more Gram-positive pathogens as key components involved in host tissue colonization; however, until 2009 these structures remained undiscovered in commensal lactobacilli (Kankainen et al., 2009).

In 2009, the presence of mucus-binding pili displayed on the surface of *L. rhamnosus *GG was described (Kankainen et al., 2009). *L. rhamnosus *GG is a probiotic bacterial strain which has been used for over two decades. Additionally, *L. rhamnosus *GG shows exemplary ability to adhere to Caco-2 cells as compared to other probiotic strains (Jacobsen et al., 1999). The genome sequence of *L. rhamnosus *GG revealed two potential clusters of pilus-encoding genes in tandem with a *srtC *gene (**Figure 2B**). The first cluster identified contained genes for *spaA* (LGG_00442), *spaB *(LGG_00443), and *spaC* (LGG_00444) clustered with *srtC_1_*(LGG_00441), while the second cluster contained genes for *spaD *(LGG_02370), *spaE *(LGG_02371), and *spaF *(LGG_02372) clustered with *srtC2* (LGG_02369; Kankainen et al., 2009). Furthermore, demonstration of the expression and presence of pilin-like structures on the surface of *L. rhamnosus *GG was accomplished using immunogold transmission electron microscopy, as was first described by Ton-That and Schneewind (2003) in *C. diphtheriae*. Double labeling, first with primary antibodies to the SpaC subunit of the pili and subsequently with a secondary antibody containing gold nanoparticles, allows the pili to be detected under transmission microscopy. Remarkably, the pili were not only identifiable, but they were relatively numerous at approximately 10–50 pili per cell (Kankainen et al., 2009).

The *spa *genes found in *L. rhamnosus *GG have been further characterized with regard to their function in assembling pili and in their ability to adhere to mucin. The protein product of the first gene following *srtC_1_*, the SpaA subunit, forms the backbone of the pili in *L. rhamnosus *GG*. *SpaB is found at the base of the pilin structure and is likely attached to the cell wall through the action of the SrtA enzyme, which is also encoded in the genome. Finally, SpaC can be found flanking the pilin shaft. SpaB, and to some extent SpaC, contribute to the adhesive capacity of *L. rhamnosus *GG to mucin through different mechanisms (Reunanen et al., 2012). Insertional inactivation of the *spaC *gene essentially abolishes binding to human intestinal mucus, and expression and further purification of this pilus subunit from *E. coli *showed significant binding to immobilized human intestinal mucus. The SpaB protein showed even the greatest degree of binding to human intestinal mucus which was attributed to its net positive charge facilitating binding with negative residues present in human mucus (von Ossowski et al., 2010).

To date, pili have not been functionally identified in other species of food-adapted or probiotic lactobacilli. Gene clusters with homologous structures to those found in *L. rhamnosus *GG have been described in *L. casei *BL23. In *L. casei *BL23, the gene cluster encoding *spaA*, *spaB*, *spaC*, and a class C sortase appear to be genetically intact; however, functional pili on the surface of *L. casei *BL23 has not been reported (Munoz-Provencio et al., 2012). Furthermore, the gene cluster encoding *spaD*, *spaE*, and *spaF *appears to be present, but with truncations in *spaE *and *spaF. *Recently, a gene encoding a sortase C homolog flanked by three genes with LPXTG motifs was identified in *L. lactis *IL1403. The expression of the genes and the formation of pili could not be detected under normal growth conditions; however, cloning and overexpression of the gene cluster under a high copy lactococcal promoter led to pili display (Oxaran et al., 2012).

## LPXTG MOTIF AND BIOTHERAPEUTIC APPLICATION IN LACTIC ACID BACTERIA

The conserved C-terminal anchor motif recognized by sortase in Gram-positive microbes has been suggested as a means of antigen display in vaccine development (Norton et al., 1996; Bermudez-Humaran et al., 2003; Cortes-Perez et al., 2003, 2005; Fredriksen et al., 2010; Kajikawa et al., 2011). Specifically, the use of the LPXTG motif has been investigated for *in vitro *vaccine delivery using food grade and probiotic lactobacilli as the presentation vector for the antigen. Unlike vaccine delivery vehicles which rely on attenuated strains of pathogenic bacteria, food grade LAB and notably probiotic lactobacilli present an alternative delivery vehicle as they have a safe history of use in foods and dietary supplements, are GRAS, and are able to survive passage through the GIT for vaccine delivery to the mucosal immune system. In addition, LAB have been shown to also have Sec and C-terminal cell wall anchoring machinery which can be exploited for antigen immobilization. To date, LAB and sortase-mediated cell wall anchoring have been explored in the display of potential vaccine antigens including tetanus toxin fragment C (TTFC; Norton et al., 1996), human papillomavirus (HPV) type 16 E7 antigen (Bermudez-Humaran et al., 2003; Cortes-Perez et al., 2003, 2005), the oncofetal antigen (Fredriksen et al., 2010), and *Salmonella enterica *serovar *typhimurium* flagellin (FliC; Kajikawa et al., 2011).

The functionality of sortase-mediated cell wall localization in LAB was demonstrated through display of the M6 protein, a LPXTG-anchored virulence factor of *S. pyogenes, *in *L. lactis*. The M6 protein was also successfully displayed on the cell wall of other LAB including *L. fermentum *LEM83, *L. sakei *23K, and *S. thermophilus *CNRZ302. This was achieved through cloning of the gene encoding the M6 protein (*emm6*) into the aforementioned LAB and then examining the distribution of the M6 protein using Western blot analysis. The authors were able to demonstrate the conserved nature of sortase anchoring among Gram-positive organisms; however, they noted differences in anchoring efficiency between different LAB. For example, M6 protein could be detected in the supernatant from the cocci, while it was not readily detected in the supernatant collected from the rod-shaped lactobacilli (Piard et al., 1997). The authors attributed the differences in successful anchoring to cell wall composition or cell wall turn over. This finding was reproduced by Dieye et al. (2001) who showed inefficient cell wall localization of their reporter protein, staphylococcal nuclease A, in *L. lactis *when it was coupled to the M6 protein cell wall anchor and signal peptide. By switching the signal peptide to one of lactococcal origin (Usp45), M6 was able to be efficiently displayed in *L. lactis *as well as in other LAB including *L. casei*, *L. sakei*, and *L. plantarum*. These studies suggest that the sortase machinery is functionally different across LAB and have the capacity to recognize substrates from an unrelated microbe (Piard et al., 1997; Dieye et al., 2001).

Demonstration of the cross functionality of LPXTG cell wall anchoring across Gram-positive species pointed to new directions in vaccine design. Localization of antigen to the cell wall had been shown to not only be effective, but also substantially increases immune responses compared to the intracellular or secreted form of the antigen (Reveneau et al., 2002). In an initial study of the effectiveness of vaccine delivery of the TTFC in *L. lactis*, anchoring of TTFC using the PrtP cell wall anchor was found to elicit the most robust anti-toxin immunoglobulin G (IgG) response as compared to the toxin expressed in the soluble form in a murine model (Norton et al., 1996). In this case, the LPXTG anchor from the PrtP gene used to couple the TTFC antigen to the cell wall was of endogenous origin, as opposed to an exogenous anchoring motif from *S. pyogenes* used to display the M6 protein in *L. lactis*. The enhanced immune response against TTFC can be attributed to successful localization of the antigen to the cell wall due to sortase specificity for the endogenous anchor.

Two oncogenes, the E7 antigen from HPV type-16 and the oncofetal antigen, have also been expressed in LAB as potential vaccine candidates (Bermudez-Humaran et al., 2003; Cortes-Perez et al., 2003, 2005; Fredriksen et al., 2010). The E7 antigen is specific to cervical cancer, while the oncofetal antigen has been found on all mammalian tumors (Fredriksen et al., 2010). In both studies, the probiotic LAB species, *L. plantarum*, was used as a model vaccine vector. As was shown in display of TTFC, successful presentation of both the E7 and oncofetal antigen was achieved using a species-specific cell wall anchor. The consensus sequence for cell wall anchoring by sortase in *L. plantarum* differs from that of LPXTG found in lactococci and streptococci as it has been shown to recognize the motif, LPQTXE (Kleerebezem et al., 2003). The use of this *L. plantarum *sortase consensus sequence did not only enhance the efficiency of surface display as indicated by Western blot, but also, in the case of oncofetal antigen, promoted IgG oncofetal-specific immune responses in mice after oral immunization (Fredriksen et al., 2010).

One issue arising from studies of the efficacy of oral administration vaccines, is that differences in IgG responses to the specific antigen were lower when lactobacilli were fed orally as opposed to administered through the nasal route (Reveneau et al., 2002). Kajikawa et al. (2011) examined this phenomenon when they engineered a recombinant strain of *L. acidophilus *NCFM expressing the *Salmonella* flagellin (FliC), which was covalently linked to the cell wall using an LPXTG motif. The goal of the study was to evaluate the potential of FliC to serve as a vaccine adjuvant for LAB vaccines; however, it can be argued that the major finding of this research was that antigen display on the cell surface is susceptible to degradation by gastric juices. In order to protect the FliC fragment on the surface of *L. acidophilus*, the recombinant *L. acidophilus *cell suspensions were supplemented with either sodium bicarbonate or soybean trypsin inhibitor (SBTI). Both of these treatments were found to protect the antigen from degradation when incubated in simulated gastric juices; however, SBTI has a greater protective effect likely due to its sequestration of bile away from the bacterial cells thus contributing to increase viability and robust antigen production (Kajikawa et al., 2011).

Taken together these studies indicate that vaccine delivery in LAB using LPXTG or LPXTG-like cell wall anchors has great potential. Additionally, these studies highlight some important considerations in the development of LAB vaccine vectors. Although cell wall anchoring and surface display functions vary across Gram-positive species, they also indicate sortase specificity for its target domain, which leads to differences in the efficiency of antigen display. The study conducted by Kajikawa et al. (2011) indicates that despite efficient surface display, protection of antigens from GIT juices may be crucial in achieving the most robust immune response. Finally, these studies further validated the presence of functional sortase proteins in LAB which operate in protein anchoring to the cell wall.

## CONCLUSION

Interest in sortase and sortase protein substrates have extended beyond the arena of pathogens and promotion of intimate associations and infection. Rather, sortase is clearly an important mechanism for display of cell surface proteins, a significant niche related trait of commensal and probiotic microbes associated with the intestinal mucosa. It is not difficult to envision further use of sortase machinery present in LAB to present cell surface oral vaccines, given the success of antigen display discussed in this review. The hypothesis that sortase enzymes may play crucial roles in bacterial physiology (as in the case of PrtP in *L. lactis *ssp. *cremoris *MG1363) as well as mediating bacterial-host interactions has accelerated the study of this enzyme in different species of LAB. The ability to access and examine sortase enzymes and their targets using genomic analysis tools has been crucial. The mechanisms of sortase action and covalent linkage of SDPs to the cell wall is a successful tactic of surface display in Gram-positive bacteria which has enabled some pathogenic organisms to gain advantage of their host, while allowing others, namely commensal and probiotic bacteria, to adhere and interact with their hosts in positive way.

## Conflict of Interest Statement

The authors declare that the research was conducted in the absence of any commercial or financial relationships that could be construed as a potential conflict of interest.

## Footnotes

1 http://www.cmbi.ru.nl/lab_secretome/index.php

2 http://www.ebi.ac.uk/Tools/pfa/iprscan/

3 http://www.cbs.dtu.dk/services/SignalP/
